# Study protocol for a phase II dose evaluation randomized controlled trial of cholecalciferol in critically ill children with vitamin D deficiency (VITdAL-PICU study)

**DOI:** 10.1186/s40814-017-0214-z

**Published:** 2017-12-08

**Authors:** Dayre McNally, Karin Amrein, Katharine O’Hearn, Dean Fergusson, Pavel Geier, Matt Henderson, Ali Khamessan, Margaret L. Lawson, Lauralyn McIntyre, Stephanie Redpath, Hope A. Weiler, Kusum Menon, on behalf of the Canadian Critical Care Trials Group

**Affiliations:** 1 Research Institute, Children’s Hospital of Eastern Ontario, 401 Smyth Road, Ottawa, ON K1H 8L1 Canada; 2 Department of Pediatrics, Faculty of Medicine, University of Ottawa, Children’s Hospital of Eastern Ontario, Ottawa, Canada; 3 Division of Endocrinology and Metabolism, Department of Internal Medicine, Medical University of Graz, Graz, Austria; 4 Department of Epidemiology, University of Ottawa and Ottawa Hospital Research Institute (OHRI), University of Ottawa, Ottawa, Canada; 5 Newborn Screening Ontario, Ottawa, Canada; 6 Euro-Pharm International Canada Inc., Montreal, Canada; 7 Department of Medicine (Division of Critical Care), Ottawa Hospital Research Institute (OHRI), University of Ottawa, Ottawa, Canada; 8 School of Dietetics and Human Nutrition, Faculty of Agricultural and Environmental Sciences, McGill University, Montreal, Canada

**Keywords:** Vitamin D, Pediatrics, Critical care, Randomized controlled trial, Vitamin D deficiency

## Abstract

**Background:**

Clinical research has recently demonstrated that vitamin D deficiency (VDD) is highly prevalent in the pediatric intensive care unit (PICU) and associated with worse clinical course. Multiple adult ICU trials have suggested that optimization of vitamin D status through high-dose supplementation may reduce mortality and improve other clinically relevant outcomes; however, there have been no trials of rapid normalization in the PICU setting. The objective of this study is to evaluate the safety and efficacy of an enteral weight-based cholecalciferol loading dose regimen in critically ill children with VDD.

**Methods/design:**

The VITdAL-PICU pilot study is designed as a multicenter placebo-controlled phase II dose evaluation pilot randomized controlled trial. We aim to randomize 67 VDD critically ill children using a 2:1 randomization schema to receive loading dose enteral cholecalciferol (10,000 IU/kg, maximum of 400,000 IU) or a placebo solution. Participants, caregivers and outcome assessors will be blinded to allocation. Eligibility criteria include ICU patient, aged 37 weeks to 18 years, expected ICU length of stay more than 48 h, anticipated access to bloodwork at 7 days, and VDD (blood total 25 hydroxyvitamin D < 50 nmol/L). The primary objective is to determine whether the dosing protocol normalizes vitamin D status, defined as a blood total 25(OH)D concentration above 75 nmol/L. Secondary objectives include an examination of the safety of the dosing regimen (e.g. hypercalcemia, hypercalciuria, nephrocalcinosis), measures of vitamin D axis function (e.g. calcitriol levels, immune function), and protocol feasibility (eligibility criteria, protocol deviations, blinding).

**Discussion:**

Despite significant observational literature suggesting VDD to be a modifiable risk factor in the PICU setting, there is no robust clinical trial evidence evaluating the benefits of rapid normalization. This phase II clinical trial will evaluate an innovative weight-based dosing regimen intended to rapidly and safely normalize vitamin D levels in critically ill children. Study findings will be used to inform the design of a multicenter phase III trial evaluating the clinical and economic benefits to rapid normalization. Recruitment for this trial was initiated in January 2016 and is expected to continue until November 30, 2017.

**Trial registration:**

Clinicaltrials.gov NCT02452762

**Electronic supplementary material:**

The online version of this article (10.1186/s40814-017-0214-z) contains supplementary material, which is available to authorized users.

## Background

Numerous observational studies have demonstrated that vitamin D deficiency (VDD) is highly prevalent in pediatric intensive care units (PICU) around the globe, with rates ranging from 30 to 80% [1–7]. These observations are concerning as vitamin D is accepted as a pleiotropic hormone important for the proper functioning of organ systems central to the progression of critical illness. VDD has been implicated in the health and stress response of the cardiovascular, immune, respiratory, and neurological systems. The biological mechanisms through which VDD may contribute to primary or secondary ICU pathophysiology have been discussed in detail elsewhere [8–10]. Multiple observational studies have established an association between lower vitamin D levels and worse clinical outcomes in critically ill children [1, 3, 4, 7, 11]. These same findings are well established in the adult intensive care unit (ICU) setting, with cumulative results on tens of thousands of critically ill adults reporting associations between hormone level and organ dysfunction, health resource utilization, and mortality [12–18]. In 2014, two systematic reviews and meta-analyses of adult ICU observational data independently concluded that a total 25 hydroxyvitamin D level, 25(OH)D, below 50 nmol/L was associated with an almost twofold increased risk of mortality [19, 20]. However, as the findings are based on data from observational studies, it is important not to draw conclusions on causality as the relationship may be driven by confounding factors.

Multiple adult and pediatric critical care research groups have hypothesized that optimization of vitamin D status could improve clinical outcomes and save health care costs [21, 22]. This optimization will require a significant change in the current approach to vitamin D supplementation. Presently, usual care involves the enteral or parenteral administration of between 200 and 1000 IU/day of cholecalciferol. Although widespread, this approach may only minimally benefit patients. Substantial literature from ambulatory populations suggests that administration of low-dose daily supplementation can require months to rebuild body stores and may not be sufficient in some patients. Moreover, emerging evidence from hospitalized and ICU patients suggest that levels often remain constant and may even decline during the first few weeks in the ICU with usual care [18, 23]. The Institute of Medicine Tolerable Upper Intake Level supports the use of higher daily doses which are age-based and range from 1000 to 4000 IU [24]. Although this would be an improvement, a recent systematic review by our group identified that these doses still require at least a month to normalize vitamin D status in those who are VDD [25]. This timeframe would be reasonable in the outpatient settings, but may not be optimal in critical illness where prolonged periods of VDD could contribute to secondary pathology, protracted recovery, and may influence long-term quality of life. Consequently, alternative approaches to vitamin D supplementation will be required to rapidly optimize vitamin D status in the ICU setting. The high VDD rates in critically ill patients, while concerning, could also present a unique opportunity to improve outcomes following the critical stages of illness once an ICU-specific approach to vitamin D supplementation is identified.

Recognizing that vitamin D is a safe, simple, and inexpensive intervention, multiple adult ICU research groups have proceeded to randomized controlled trials (RCT) [26–33]. Of these, two RCTs have been especially important in demonstrating the benefit of vitamin D supplementation in ICU. Both trials sought to rapidly normalize vitamin D status in ICU, over 2 to 5 days, using enteral loading dose cholecalciferol therapy. VITdAL-ICU, the only phase III trial to date, reported that administration of a single enteral cholecalciferol loading dose (540,000 IU) to VDD critically ill adults reduced mortality in those with severe deficiency and improved long-term functional outcomes in those with more moderate deficiency [27]. Second, the RCT by Han and colleagues comparing 250,000 and 500,000 IU enteral cholecalciferol (divided over 5 days) with placebo in critically ill ventilated adults reported a statistically significant reduction in length of stay [26]. Two adult systematic reviews with meta-analyses published in late 2016 suggested that high-dose vitamin D has the potential to reduce ICU mortality and length of stay [15, 16].

Although encouraging, the findings from adult trials are not directly transferrable to the PICU setting and clinical trials will be required to address the unique characteristics of children. Primary among the issues to be resolved include establishing a dosing regimen that safely allows for rapid normalization of vitamin D status across a broad range of pediatric ages and conditions. We recently performed a systematic review and meta-regression of pediatric high-dose vitamin D trials to help inform the dosing regimen for a rapid normalization RCT in the PICU setting [25]. This analysis identified weight-based enteral loading therapy of 10,000 IU/kg, up to a maximum of 400,000 IU, as the dosing regimen most likely to rapidly and safely normalize levels (in unwell VDD children). Although not commonly used in North America, intermittent or loading dose cholecalciferol is well studied for the prevention and treatment of rickets in many countries and is included in reviews and positions statements [34, 35]. That said, as concern persists about the safety of high-dose vitamin D, we completed a meta-analysis of clinical trial data which failed to identify evidence of toxicity until doses generated group average 25(OH)D level above 200 nmol/L (consistent with dosing above 400,000 IU). The dosing regimen identified in our meta-analysis should avoid the supraphysiological concentrations (> 200 nmol/L) associated with toxicity in the majority of patients. While data from this extensive review support the efficacy and safety of this dose, rigorous clinical trials are required before it can become part of standard care in PICU.

In collaboration with the Canadian Critical Care Trials Group (CCCTG), we propose a placebo-controlled phase II dose evaluation pilot trial of a weight-based enteral loading dose of cholecalciferol in critically ill children with vitamin D deficiency. The primary objective of the VITdAL-PICU pilot study is to determine whether the dosing regimen can rapidly normalize vitamin D levels in critically ill children with VDD. Secondary objectives are to evaluate the safety of the dosing regimen (e.g., hypercalcemia and hypocalcemia), improved vitamin D axis functioning (e.g., active hormone levels and calcium metabolism), and differences in blood measures of inflammation and innate immunity (e.g., C-reactive protein and procalcitonin) compared to placebo. Tertiary feasibility objectives are to assess adherence to the treatment protocol (including blinding), assess the appropriateness of our eligibility criteria for the full trial, and estimate the rate of patient recruitment while understanding barriers to recruitment.

## Methods/design

This study was designed as a placebo-controlled dose evaluation pilot RCT. Although the placebo group is not directly relevant to our primary objective of dose evaluation, this study design is essential to allow us to address our secondary and tertiary objectives. The inclusion of a placebo group will allow us to describe blood or urine calcium levels (i.e., surrogates of vitamin D toxicity) for children who are and are not exposed to the rapid restoration dose. Further, the placebo arm is required to properly evaluate recruitment and our ability to achieve blinding. The study protocol adheres to the Standard Protocol Items: Recommendations for Interventional Trials (SPIRIT) checklist (see checklist, Additional file 1). A summary of the protocol is provided in Table 1.

**Table 1:** World Health Organization trial registration data set––structured summary

Data category	Information
Primary registry, trial identifying #	Clinicaltrials.gov identifier-NCT02452762
Date of registration in primary registry	March 15, 2015
Secondary identifying numbers	Health Canada control number and protocol title #184825, VITdAL-PICU-01; Children’s Hospital of Eastern Ontario REB number 15/18E
Sources of monetary support	AHSC Innovation Fund at the Children’s Hospital of Eastern Ontario, Canadian Health Research Institutes Project Scheme Grant
Primary sponsor	Investigator initiated: James Dayre McNally Children’s Hospital of Eastern Ontario 401 Smyth Road Ottawa, Ontario K1H 8L1 Phone: 613-737-7600 ext. 3553 Email: dmcnally@cheo.on.ca
Secondary sponsor	Children’s Hospital of Eastern Ontario Research Institute
Contact for public queries	JDM, Pediatric Critical Care, Children’s Hospital of Eastern Ontario, Canada
Contact for scientific queries	JDM, Pediatric Critical Care, Children’s Hospital of Eastern Ontario, Canada
Public title	Rapid normalization of vitamin D in critically ill children: a phase II dose evaluation randomized controlled trial (VITdAL-PICU)
Scientific title	Rapid normalization of vitamin D in critically ill children: a phase II dose evaluation randomized controlled trial
Country of recruitment	Canada, Chile, Austria
Health problem under investigation	Vitamin D deficiency in critically ill children
Key inclusion and exclusion criteria	*Inclusion criteria*: admitted to ICU, corrected gestational age > 37 weeks and < 18 years, expected ICU admission in excess of 48 h and expected to have access for bloodwork at day 7, blood 250HD < 50 nmol/L *Exclusion criteria*: significant gastrointestinal disorder preventing enteral drug administration, hypercalcemia, confirmed or suspected William’s syndrome, nephrolithiasis or nephrocalcinosis, imminent plan for withdrawal of care or transfer to another ICU, physician refusal, previous enrollment in the study, patient known to have Granulomatus disease, severe liver dysfunction/liver failure, hypersensitivity or allergy to vitamin D or any of the non-medical ingredients in the formulation, patient on thiazide diuretics who is also receiving regular ongoing calcium supplementation, pregnancy, digoxin-therapy
Study type	Randomized, double-blind phase II dose evaluation trial
Date of first enrollment	29 January 2016
Target sample size	67
Recruitment status	Recruitment initiated on 11 January 2016; recruitment ongoing
Primary outcome	To determine whether a weight-based enteral dosing protocol can rapidly normalize vitamin D status in critically ill deficient children

### Study setting

The VITdAL-PICU pilot study was initially designed to recruit patients from the pediatric and neonatal ICUs at a single academic center in Ottawa, Canada (Children’s Hospital of Eastern Ontario (CHEO)). To increase feasibility and further evaluate the study protocol, two additional study sites were added: Graz, Austria (Medical University of Graz) and Concepción, Chile (Hospital Guillermo Grant Benavente).

### Patient enrollment and consent

Study staff will screen pediatric ICUs (and neonatal ICU at Ottawa site) from Monday to Friday for patients meeting eligibility criteria. Eligibility criteria include patient admitted to the ICU, corrected gestational age > 37 weeks to age < 18 years, expected ICU admission in excess of 48 h and likely to have access for bloodwork at day 7, and blood 25(OH)D level of < 50 nmol/L (regardless of prior approach to supplementation). The inclusion and exclusion criteria, and justification for each criterion, are presented in detail in Table 2. If a patient meets all eligibility criteria, with the exception of 25(OH)D level, the study team will approach the legal guardian for informed consent. If consent is obtained, the patient’s vitamin D status will be determined, and the patient randomized into the study if they meet the 25(OH)D criteria.

**Table 2:** VITdAL-PICU pilot study inclusion and exclusion criteria

Inclusion criteria	Justification
Admitted to ICU	Admitted to the pediatric or neonatal ICU. The neonatal ICU was included to improve recruitment and to increase the number of participants aged < 30 days. At randomization, patients are stratified by recruitment site (PICU vs NICU) to account for site-specific practice variation.
Corrected gestational age > 37 weeks to age < 18 years	Pediatric study (< 18 years). Very premature infants are at significantly increased risk for nephrocalcinosis [40, 41]; children < 37 weeks corrected gestational age are excluded to reduce the risk of enrolling patients with an increased chance of adverse outcome.
Expected ICU admission in excess of 48 h and likely to have access for bloodwork at 7 days of hospital stay (determined by medical team)	This inclusion criterion was selected to (i) include an ICU population with a higher illness severity and (ii) to increase the likelihood of obtaining a blood sample at day 7 for measurement of the primary outcome.
25(OH)D level < 50 nmol/L	Although thresholds and terminology can vary, vitamin D deficiency is generally accepted as < 50 nmol/L (severe deficiency as < 30 nmol/L) [42–45]. Multiple observational ICU studies have shown an association with poorer outcome once vitamin D levels fall below 50 nmol/L [19, 20]. Some evidence exists that shows the benefit of supplementation (as defined by mortality) may be limited to ICU patients with severe deficiency (< 30 nmol/L) [2, 27]. In those with more moderate deficiency (30–50 nmol/L) benefits were observed in repeated infections and quality of life [2, 27]. To evaluate this in PICU, we will compare changes in biochemistry and clinical measures separately for the groups with starting 25(OH)D above and below 30 nmol/L.
Exclusion criteria	Justification
Significant gastrointestinal disorder preventing enteral drug administration	At present, there is no intravenous form of cholecalciferol, preventing the inclusion of ICU patients who cannot receive enteral drugs.
Hypercalcemia, excluding transient abnormalities and those related to parenteral calcium administration for hypocalcemia	Patients presenting with hypercalcemia may be at increased risk for toxicity or adverse outcome with a significant abrupt increase in vitamin D level.
Confirmed or suspected William’s syndrome	Patient with William’s syndrome have a genetic susceptibility to hypercalcemia, and current guidelines recommend against any vitamin D supplementation [46, 47].
Patient know to have nephrolithiasis or nephrocalcinosis	Patients presenting with nephrolithiasis or nephrocalcinosis would be at increased risk for an adverse outcome.
Imminent plan for withdrawal of care or transfer to another ICU	If a patient will be transferred or have care withdrawn imminently, we would be unlikely to obtain either day 3 or day 7 blood and urine sample (25OHD, hypercalcemia, hypercalciuria).
Physician refusal	If the treating physician has concerns about study participation (e.g., patient presents with clinical symptoms of severe vitamin D deficiency and treating physician plans to administer a large dose of vitamin D), the patient would not be randomized
Previous enrolment in the VITdAL-PICU pilot study	Patients who previously participated in the VITdAL-PICU study will be excluded to avoid confounding.
Patient known to have granulomatous disease (tuberculosis or sarcoidosis)	Excess active vitamin D hormone has been observed in patients with granulomatous diseases (tuberculosis/sarcoidosis) and can potentially lead to hypercalcemia or hypercalciuria through increased intestinal absorption of calcium [48].
Severe liver dysfunction/failure	The liver plays an essential role in vitamin D metabolism. Patients with severe liver dysfunction/failure have a minimized capacity to convert vitamin D to its active form. To allow for a study population with a more homogenous ability to metabolize and benefit from vitamin D supplementation, patients with severe liver dysfunction/failure will be excluded.
Patient known to have hypersensitivity or allergy to vitamin D or any of the non-medicinal ingredients of the formulation	Listed as a contraindication in the product monograph
Patient on thiazide diuretics who is also receiving regular ongoing calcium supplementation above the daily recommended intake for reasons other than hypocalcemia	There is an increased risk of hypercalcemia if vitamin D is co-administered with both thiazide diuretics and calcium (as per product monograph).
Adolescent female of child-bearing age with a positive pregnancy serum test	Maternal hypercalcemia, possibly caused by excessive vitamin D intake during pregnancy, has been associated with hypercalcemia in neonates, which may lead to adverse effects (as per product monograph).
Patient on digoxin therapy	Vitamin D should be used with caution in patients on digoxin. Hypercalcemia (which may result with vitamin D use) may precipitate cardiac arrhythmias (as per product monograph).

As clinical testing and reporting of blood 25(OH)D concentration frequently requires 10 to 14 days at CHEO, rapid determination of vitamin D levels will be performed using a FDA and Health Canada approved device (FastPak®, Qualigen, Inc.). Prior to the initiation of recruitment, the FastPak® was validated, and a proficiency certificate was obtained from the Vitamin D External Quality Assessment Scheme (DEQAS) group for meeting the performance targets set by their advisory panel for 25(OH)D assays (Additional file 2). The laboratories in Austria and Chile are capable of rapid measurement of vitamin D (< 24 h); therefore, the screening sample will be analyzed by the laboratory using a validated assay.

### Randomization

Patients will be randomized 2:1 and stratified by (i) patient age (above or below 30 days of age) and (ii) recruitment location (CHEO NICU, CHEO PICU, Austria, or Chile). A 2:1 randomization (high dose: placebo) schema was employed because the control group is not directly pertinent for our primary objective of dose evaluation but required to assess our secondary objectives. The secondary objectives can be properly assessed with a smaller control group. Therefore, in order to avoid enrolling more participants than necessary, and to improve the feasibility of this dose evaluation study by minimizing costs, the decision was made to use 2:1 randomization. We have decided to stratify by age as neonates can respond uniquely to medications due to different water/fat content, hepatic, and renal functioning. Further, calcium homeostasis and the definition of abnormal for both hypercalcemia and hypercalciuria are different for neonates. Stratification by neonatal status will ensure that these differences are equally distributed between the two groups.

### Allocation concealment and blinding

The Methods Centre at the Ottawa Hospital Research Institute will prepare a computer-generated randomization list. Randomized allocation will be achieved as follows: At enrolment, study staff will record the next available study ID number within the correct stratification group on the study drug order form. The pharmacist will match the study ID number assigned to the hard-copy randomization list to determine treatment allocation and will dispense the appropriate study drug and dose.

Blinding during this pilot study is necessary to allow us to evaluate the feasibility of a phase III trial designed to evaluate clinically relevant outcomes that are subjective in nature. Study personnel (study coordinator, research assistants, principal investigator, co-investigators, site investigators, data management personnel, and statisticians), members of the health care team (treating physicians, bedside nurses, and clinical pharmacists), and patients/families will be blinded to the study group assignment. It is possible that the study safety officer (PG) will become aware of study group allocation. The safety officer, who does not contribute to the medical care of the patients, will review each study participant’s vitamin D level from the day 7 blood sample to identify participants at potential risk for vitamin D toxicity (25(OH)D > 200 nmol/L) in real-time. Since it is expected that the vitamin D level of patients in the control group will not increase significantly from the time when they met eligibility criteria (25(OH)D < 50 nmol/L), the safety officer will likely be able to determine which patients were allocated to the vitamin D treatment arm if their discharge vitamin D level is substantially higher than 50 nmol/L. Otherwise, the assigned intervention will not be revealed until all patients have been discharged from hospital (censored at 90 days), determination of research-related biochemical testing is complete, and the research database has been finalized. To maintain blinding, the randomization lists will only be accessible to the Data Management Services of The Methods Centre at the Ottawa Hospital Research Institute and to the research pharmacist(s). Further, the active drug and placebo will be identical in appearance, consistency, volume, taste, and smell.

In the event of an unexpected serious adverse event that is believed to be related to vitamin D administration, blinding can be broken at the request of clinical service. The randomization code and list of randomized participants will be stored at the pharmacy. Clinical service will notify the principal investigator, who will then contact the pharmacy to unblind the participant. In the event that a participant’s day 7 25(OH)D level is > 200 nmol/L, the safety officer will refer the patient to endocrinology. The patient will be managed clinically as determined by endocrinology, who may elect to unblind the participant.

### Interventions

**Fig. 1:**
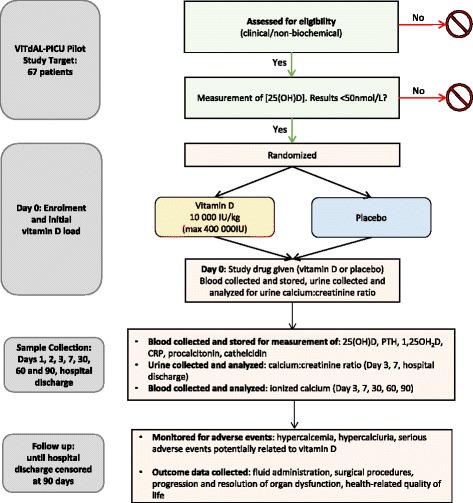
Protocol flow diagram for the VITdAL-PICU pilot study

Participants randomized to the experimental arm (see protocol flow diagram, Fig. 1) will receive an enteral cholecalciferol load (vitamin D3 (cholecalciferol) oral solution 50,000 IU/mL, Euro-Pharm International Canada Inc.) at a dose of 10,000 IU/kg (maximum 400,000 IU). This dose was chosen based on our systematic review of all pediatric trials reporting on the administration of high-dose vitamin D [25]. This review comprehensively evaluated the ability of different dosing regimens to rapidly normalize vitamin D levels and concluded that a weight-based enteral loading therapy of approximately 10,000 IU/kg was the suitable dosing regimen to rapidly normalize vitamin D status.

Participants randomized to the control group will receive a placebo solution equivalent in volume to the appropriate dose of cholecalciferol. The placebo will be provided by Euro-Pharm International Canada Inc. and contains caramel color, cherry flavor, citric acid (anhydrous), glycerin, polysorbate 80, propylene glycol, purified water, and sucralose. The control product will be identical in smell, taste, and appearance to the study drug. Sites in Austria and Chile will use vitamin D and placebo from Fresenius Kabi (Oleovit D_3_) and Laboratorios Andromaco SA (D’Vidamax 50,000 IU Oral Solution), respectively.

At the discretion of the health care team, study participants in either arm can receive routine or standard of care daily vitamin D administration. If the health care team chooses to prescribe vitamin D as part of clinical care, the study team will recommend a dose of 400–800 IU/day. However, the health care team will be free to prescribe doses outside of this range, and it will not be documented as a protocol violation. If a patient presents with symptoms of severe vitamin D deficiency and the treating physician intends to administer a large dose of vitamin D, or if standard treatment includes administration of high-dose vitamin D (i.e., patient admitted with severe burns) the patient will not be enrolled in the study (excluded due to physician refusal). Additional vitamin D administration will be documented on the case report form. With the exception of study drug administration, there will be no other changes to clinical management. Co-interventions will not be protocolized as the study is blinded, and any differences should relate to either random chance or effects from the study drug. Major co-interventions will be captured on the case report form during data collection.

### Blood and urine sampling

Blood samples will be collected at the time of clinically indicated venipuncture or through arterial or central venous line access. When possible, blood will be collected at enrolment, and on days 1, 2, 3, 7, 30, 60, and 90 and at hospital discharge. Urine samples will be collected at enrolment, on days 3 and 7 and at hospital discharge. Blood samples at each time point will be analyzed for 25(OH)D levels. Ionized calcium will be measured on days 3, 7, 30, 60, and 90, unless ionized calcium has been measured through clinical bloodwork in the preceding 24 h, or a clinical calcium sample is planned for that day. Urine samples at each time point will be analyzed for calcium:creatinine ratios.

### Outcomes

Our primary outcome is the proportion of critically ill children who achieve blood 25(OH)D concentration above 75 nmol/L by day 7 (normalization of vitamin D status). 25(OH)D is widely regarded as the best indicator of vitamin D status [36]. If bloodwork cannot be obtained on day 7 ± 48 h (e.g., no access to bloodwork, patient discharged), the patient’s 25(OH)D level from day 3 will be used for the primary outcome. Results from an adult ICU RCT evaluating a single 540,000 IU enteral load demonstrated no difference in 25(OH)D levels at day 3 versus day 7 [27]. Secondary and tertiary outcome measures are summarized in Table 3 and include an examination of the safety of the vitamin D dosing regimen, vitamin D axis function, immune function, feasibility, and identification of phase III trial outcomes.

**Table 3:** Secondary and tertiary outcomes for the VITdAL-PICU pilot study

Outcome	Explanation of outcome analysis criteria
Vitamin D-related adverse events	We will evaluate for potential toxicity using two well-accepted surrogate outcome measures: • Hypercalcemia—defined as an ionized calcium level above 1.40 mmol/L (children under 8 weeks as > 1.45 mmol/L). • Hypercalciuria—defined using age-specific norms and thresholds for calcium-creatinine ratios (see Table 4) We will also evaluate for, and report on, the occurrence of serious adverse events that could potentially be related to vitamin D.
Vitamin D axis function	Evaluated through an analysis of blood calcium, parathyroid hormone, and 1,25(OH)2D.
Immune function	Evaluated through an analysis of inflammatory markers (C-reactive protein, procalcitonin) and antimicrobial peptide levels (cathelicidin).^a^
Feasibility outcomes	Protocol non-adherence: protocol adherence will be considered successful if (a) major protocol deviations occur with regard to study drug administration or safety procedures in < 20% of enrolled patients. *Study drop out:* a study dropout rate of < 10% will be considered acceptable. *Evaluation of the proposed eligibility criteria*: defined as our ability to predict ICU stay longer than 48 h and bloodwork access at 7 days. Patient accrual rate: defined as the number of patients enrolled over a 2-year period. We will consider the patient accrual rate acceptable if we are able to successfully enroll 67 patients within 2 years. Ability to maintain blinding: ability to maintain blinding will be considered successful if the frequency of unblinding requests from the clinical care team and from the pharmacy is < 10%.
Phase III trial outcomes	We will assess potential outcomes for a phase III trial to better inform sample size for subsequent phases of this research program. The phase III trial outcomes that will be assessed are (i) multiorgan dysfunction (PELOD-2 score; days 0, 3, 7, and every 30 days until discharge or 90 days), (ii) readiness for PICU discharge, and (iii) quality of life measure (PedsQL™)

^a^Dependent on receiving additional funding

Specific criteria must be met to establish the feasibility of proceeding to a phase III trial. Protocol adherence will be considered successful if major protocol deviations with regard to study drug administration (e.g., patient randomized but drug not administered, drug administered late) or safety procedures (e.g., referral not given for patient who met safety criteria for referral to nephrology/endocrinology) occur in < 20% of enrolled patients. In our sample size calculation, we estimated a dropout rate of 5%; however, we will consider a study dropout rate of up to 10% as acceptable. Recruitment rate will be defined as the number of patients enrolled over a 2-year period. We will consider the patient accrual rate successful if we are able to enroll 67 patients within 2 years (average 2.5 patients/month). We will consider our ability to maintain blinding successful if the clinical care team or pharmacy request unblinding in < 10% of enrolled patients. Finally, we will also evaluate the proposed eligibility criteria, specifically, out of ability to predict ICU stay longer than 48 h and bloodwork access at 7 days. The purpose of these criteria is to ensure we are able to collect a blood sample for the primary outcome. We will consider these criteria acceptable if we are able to obtain a blood sample in 95% of enrolled patients.

### Safety procedures

Adverse events will be monitored and reported according to regulatory and local ethics board requirements. Specifically, three serious adverse events (SAEs) have been identified that would be considered both unexpected and potentially related to study drug and would require reporting Health Canada, European Medical Association and local research ethics boards. They are (i) gastrointestinal bleeding (requiring blood transfusion) and perforation (requiring surgery) within 48 h of study drug administration; (ii) persistent hypercalcemia (> 24 h in the absence of parenteral calcium administration) with renal failure requiring dialysis, nephrocalcinosis, hemodynamically significant arrhythmia, and cardiorespiratory arrest or death; and (iii) new or worsening hypercalciuria with nephrolithiasis, renal failure leading to dialysis or death. Ionized calcium levels and urine calcium:creatinine ratios from study samples will be monitored in real time by the site investigator for hypercalcemia and hypercalciuria (see Table 4 for established thresholds). Additionally, the 25(OH)D level at day 7 will be reviewed by the study nephrologist (PG). Safety procedures have been protocolized, and patients with abnormal research samples will be referred to nephrology or endocrinology as outlined in Table 4.

**Table 4:** Definition of vitamin D-related adverse events monitored in real-time through lab values from the clinical laboratory and safety procedures for elevated lab values in the VITdAL-PICU pilot study

Adverse event	Study thresholds	Definition	Safety procedures
Hypercalcemia	Ionized calcium level: > 1.40 mmol/L > 1.45 mmol/L for children < 8 weeks of age	Persistent hypercalcemia for > 24 h in the absence of calcium administration	Endocrinology consult, managed clinically as determined by endocrinology
Hypercalciuria (calcium:creatinine ratio)	Age-based thresholds: < 1 2.2 mol/mol 1–2 1.5 mol/mol 2–3 1.4 mol/mol 3–5 1.1 mol/mol 5–7 0.8 mol/mol 7–17 0.7 mol/mol	Hypercalciuria, as determined by a calcium:creatinine ratio above the study threshold in two sequential urine samples (excluding enrolment sample)	Nephrology consult, case reviewed to determine the need for repeat urine sample, abdominal ultrasound, and/or clinical management by nephrology
Hypervitaminosis D	25(OH)D > 200 nmol/L	25(OH)D level > 200 nmol/L in day 7 sample	Endocrinology consult, managed clinically as determined by endocrinology

### Sample size

The goal of our weight-based loading protocol is to achieve target 25(OH)D concentrations above 75 nmol/L in 75% of the participants who receive loading dose vitamin D. Further, the minimal acceptable proportion achieving target where we would consider proceeding with a phase III trial is 50%. This minimal acceptable proportion is based on results from the VITdAL-ICU phase II study in adult ICU that suggested a 7% absolute risk reduction in mortality with only 52% of enrolled patients achieving a 25(OH)D level > 75 nmol/L [27]. Assuming that the true proportion achieving target is 75%, a random sampling of 36 patients has ~ 90% power to return an estimate in excess of 66%––two thirds of participants achieving target 25(OH)D. Given an estimate in excess of 66% and a sample size of 36, the lower 95% confidence interval will exclude 50%. To account for 5% dropout or missing samples, we will recruit up to 40 patients into the high-dose arm, and 20 in the placebo arm, for a total sample size of 60 patients.

The assumption that 75% of patients who receive the loading dose will achieve target levels is based on our systematic review and meta-regression [25]. We anticipate that 10,000 IU/kg will raise the vitamin D level by 70 nmol/l. Assuming a group average value of 40 nmol/L in the intervention arm, this will result in a post-load group 25(OH)D level of approximately ~ 110 nmol/L. Given a standard deviation of no more than 35 nmol/L, we would anticipate that 16% of the group will not achieve a post-load value of 75 nmol/L (*z* score of − 1). However, given that some of the older children (weight above 40 kg) will receive less than 10,000 IU/kg, we have reduced our estimate of proportion achieving target to 25%.

### Recruitment, compliance, and follow-up

Based on (i) the annual ICU admission rate at participating sites, (ii) our experience with vitamin D RCTs and published work on factors affecting consent rate in PICU studies in Canada (anticipated consent rate ~ 50%) [37], (iii) discussions with the CCCTG, and (iv) the anticipated frequency of VDD in PICU, we have planned for up to 2 years as a feasible timeframe for recruitment into this dose evaluation study.

Follow-up for the VITdAL-PICU pilot study ends at 90 days, hospital discharge, or death. Consequently, loss to follow-up will primarily relate to participant withdrawal or dropout and is anticipated to be negligible (< 5%). As most critically ill ICU patients have regular blood work, we anticipate that in the absence of death, there will be research blood and urine collected for 25(OH)D and calcium determination on the vast majority (> 95%) of patients on day 7 (or day 3). For our recently completed RCT of pre-operative supplementation in children with congenital heart disease (NCT01838447), post-operative collection of research blood for the primary outcome, collected at the time of PICU admission following surgery, was 100% [38].

### Data collection and management

Data for each study patient will be entered by site study staff directly into the web-based case report form (eCRF), using only the study ID number to identify the record. The case report form has been developed and managed using an electronic data capture tool, Research Electronic Data Capture (REDCap) [39], which will be hosted at the CHEO Clinical Research Unit. REDCap is a secure, web-based application designed to support data collection for research studies. Pre-defined ranges for all data values will be set up in this application to allow data entry personnel to validate data as soon as they are entered and send data queries immediately. Missing data will be similarly managed.

The following information will be collected: demographic information, fluid and inotrope administration, surgical procedures, progression and resolution of organ dysfunction (e.g., PEdiatric Logistic Organ Dysfunction-2 score), occurrence of adverse events, length of mechanical ventilation, length of ICU and hospital stay, survival status, renal function, gastrointestinal bleeding, quality of life scores (e.g., PedsQL™ Pediatric Quality of Life Inventory™), and laboratory results. Additional information will be collected for patients who are discharged from hospital before 90 days including hospital readmission rate and frequency of illness.

Participant-related information including eCRFs and laboratory specimens will be kept strictly confidential. All records will be kept in a secure, locked location, and only research staff will have access to the records. The REDCap study database is securely protected and encrypted. All computerized databases will identify subjects by numeric codes only.

### Statistical analysis

Analyses will be performed using SAS® software (Cary, NC, USA), and a *p* value less than 0.05 will be considered statistically significant. Additionally, both treatment arms will be described using descriptive statistics: (i) means with standard deviations or medians with inter-quartile range values for continuous variables or (ii) frequencies with percentages for categorical variables.

The primary analysis for the study will be the proportion of participants in the treatment arm achieving 25(OH)D levels above 75 nmol/L on day 7 (with its 95% confidence interval). We expect 0% of the control arm to achieve the target 25(OH)D level of 75 nmol/L. This assumption is based on the fact that the control arm will receive no vitamin D treatment, or usual care vitamin D (if prescribed by the care team) which our meta-analysis demonstrated would not be able to move vitamin D levels from < 50 nmol/L to ≥ 75 nmol/L within 7 days [25]. Further, vitamin D levels of hospitalized children have been shown to stay the same or even fall over time with usual care [18, 23]. The analysis will evaluate the data using both intention to treat (primary) and per protocol (secondary) approaches. In addition, we will also report on (i) the distribution of 25(OH)D levels on day 7 using means/medians with the appropriate measure of distribution (standard deviation, IQR) and (ii) the number of study participants who developed 25(OH)D levels in excess of 200 nmol/L.

Analyses of secondary outcomes will focus on confidence interval generation. Secondary outcomes will be described by group as means with standard deviations or medians with inter-quartile range values for continuous variables, or as frequencies with percentages for categorical variables (e.g., hypercalciuria, hypercalcemia, nephrocalcinosis). We will also conduct some exploratory evaluations between groups based on data type. Outcome measures that are continuous will be evaluated using the *t* test or Mann-Whitney (where appropriate). Binary secondary outcome measures will be compared between the two treatment groups using Fisher’s exact or chi-square test. If randomization does not lead to equal distribution of important variables (e.g., weight), the above analysis will be expanded to regression modeling to allow for adjustment. Results of between group analyses will be considered hypothesis generating.

We will describe 25(OH)D and other biochemical outcomes within three different subgroups. First, we will describe 25(OH)D response in the group of patients under and over 40 kg. Second, we will describe changes in biochemistry and clinical measures separately for the groups with an enrolment 25(OH)D level above and below 30 nmol/L. Third, we will describe 25(OH)D response and clinical measures separately for newborns (age < 30 days) versus older children (age > 30 days).

### Monitoring

The Data Safety Monitoring Board (DSMB) will be comprised of two clinical experts (critical care, nephrology) and a biostatistician and will function independently from the study investigators and the steering committee. The DSMB will review safety data, blinded in aggregate and by study group, after 30 and 53 patients have completed day 7. Specifically, the DSMB will review the occurrence of hypervitaminosis D (25(OH)D > 200 nmol/L), vitamin D-related adverse events (hypercalciuria, hypercalcemia), and serious adverse events potentially related to study drug. The DSMB chair will also receive immediate notification and reports of serious adverse events determined to be related to the study drug. In addition, the DSMB chair will receive a report for all deaths and all requirements for renal replacement therapy, regardless of whether they are thought to be related to the VITdAL-PICU pilot study. There would be concern regarding the dosing protocol if more than 10% of participants achieved 25(OH)D levels above 250 nmol/L (even in the absence of clinical sequelae). If this criterion was met, a downward adjustment of the loading dose would be considered.

 Study monitoring at CHEO will be conducted by qualified peer-to-peer monitors. The monitoring review will occur approximately every 3 months or as needed based on enrollment. The principal investigator and the study coordinator will review the study regularly and assess evaluations of patients’ eligibility, study data, and adverse events in the study database. Study monitoring of sites in Austria and Chile will be performed remotely by the study coordinator and principal investigator.

### Ethics and regulatory requirements

Research ethics board approval will be obtained before initiation of recruitment. Protocol amendments will be communicated as necessary to those involved in the study. In Canada, the study protocol was approved by the Children’s Hospital of Eastern Ontario Research Ethics Board (REB reference number 15/18E), and regulatory approval was obtained from Health Canada (control number 184825). In Austria, the study protocol was approved by the Medical University of Graz Ethics Committee (REB reference 28-619 ex 15/16) and regulatory approval was obtained from European Medical Association (EudraCT number 2016-002459-38). The Chilean site obtained approval from the Ethical Scientific Committee of the Concepción Health Service (REB reference 16-03-10). Regulatory approval was not required in Chile. The study was first posted on Clinicaltrials.gov on 05/152/2015 (NCT02452762).

### Close out

At study close out, study data, source documentation, and regulatory files will be securely stored for 25 years. The study drug will be destroyed and reconciled at each site according to site standard operating procedures and regulatory requirements. Study data will only be accessible to the principal investigator, research coordinator, or delegate.

### Dissemination and access to data

The principal investigator will write up the results of the study for publication in a peer-review journal. Interim and final results will be presented at national and international scientific meetings. In addition, the CCCTG will be updated on the project and progress biannually at their scientific meetings, and other team members sharing the results at their professional meetings, outside of the critical care discipline. A summary of the study results will be prepared in lay terms and sent by mail to study participants. If funding allows, other knowledge translation methods will be explored, including a decision aid to help families make informed choices around testing vitamin D levels and the options for clinical management of the results, and media tools, pamphlets and posters directed at the general public, ICU patients and families, and physicians.

Data analysis will occur at CHEO RI/OHRI. Participant level trial data will not be sent to other sites or researchers. Co-authors may suggest sub-studies and will be given the opportunity to take the lead on manuscript preparation. Data analysis will take place at CHEO RI/OHRI and aggregate data corresponding to what is standard for a manuscript will be provided to the co-author. If participant level data is required, then a data sharing agreement will be obtained between institutions.

## Discussion

High-prevalence rates of VDD in pediatric ICUs worldwide and the recognized importance of vitamin D to the health of multiple organ systems suggest that rapid normalization could represent a simple, inexpensive, and safe means of improving outcomes and reducing health care spending. An important step towards determining the potential benefit of rapidly normalizing vitamin D status in PICU is the establishment of an evidence-based dosing protocol. This phase II dose evaluation study aims to determine the effectiveness and safety of a weight-based enteral rapid restoration dose of vitamin D in deficient critically ill children and is the first step towards a definitive phase III clinical trial to evaluate both the clinical and economic benefits of rapid normalization in this population.

To facilitate the success of completing this pilot RCT and the subsequent phase III trial, ongoing review, problem identification, with adjustments are imperative. Since initiating recruitment, the authors have implemented a number of modifications to the protocol aimed at increasing feasibility. Although the majority have been minor, a significant change was made to the dosing regimen after 5 months of recruitment (12% of target sample size given two doses). The original protocol included a second loading dose of cholecalciferol on day 3 (if the 25(OH)D level measured on day 3 was not above 75 nmol/L). The second dose was initially included due to concerns that some critically ill children might not achieve target 25(OH)D levels with a single dose, as observed in the similar adult VITdAL-ICU trial. The decision to drop the second dose was based on several factors, including (i) a literature update suggesting that the second load may not be necessary, and evidence suggesting clinical benefit with a single enteral load [26, 27]; (ii) concern from other sites suggesting it may prevent their participation; (iii) cost of additional drug and measuring 25(OH)D status on day 3 to determine dosing level; (iv) feedback from participants and potential participants that the study protocol, specifically the day 3 dose, was complicated and difficult to understand which the authors felt may have a negative impact on recruitment rate; and (v) although procedures were put in place to maintain blinding during measurement of 25(OH)D at day 3, the second dose increased the chances of accidental unblinding. Additional, more minor changes, to the protocol are presented and discussed in Additional file 3.

### Trial status

Recruitment for the VITdAL-PICU pilot study started at the Children’s Hospital of Eastern Ontario in January 2016. Although initially started as a single center RCT in the PICU, recruitment was expanded to sites in Chile and Austria on May 1, 2017. At the time of manuscript acceptance, approximately 95% (*n* = 64) of the target sample size has been enrolled. Recruitment is anticipated to continue until November 30, 2017.

## Additional files

Additional file 1: PIRIT checklist. Completed SPIRIT checklist. (PDF 60 kb)

Additional file 2: Proficiency of 25(OH)D analysis. Certificate of proficiency from D E Q A S (Vitamin D External Quality Assessment Scheme) for analytical reliability of 25 hydroxyvitamin D (25(OH)D). (PDF 70 kb)

Additional file 3: Table. Description of minor protocol changes made during the conduct of the study (protocol versions 1 through 9). (PDF 82 kb)

## Abbreviations

25(OH)D: 25-Hydroxyvitamin D
CHEO: Children’s Hospital of Eastern Ontario
DSMB: Data Safety Monitoring Board
eCRF: Electronic case report form
ICU: Intensive care unit
IU: International units
NICU: Neonatal intensive care unit
PELOD: Pediatric Logistic Organ Dysfunction
PICU: Pediatric intensive care unit
RCT: Randomized controlled trial
REB: Research ethics board
