# Supplementary material for: Study protocol for a phase II dose evaluation randomized controlled trial of cholecalciferol in critically ill children with vitamin D deficiency (VITdAL-PICU study)
Source: Pilot Feasibility Stud. 2017 Dec 8;3:70. doi: 10.1186/s40814-017-0214-z (PMC5721544; doi:10.1186/s40814-017-0214-z)
Supplement: Supplementary file 2 — Proficiency of 25(OH)D analysis. Certificate of proficiency from D E Q A S (Vitamin D External Quality Assessment Scheme) for analytical reliability of 25 hydroxyvitamin D (25(OH)D). (PDF 70 kb) [file 40814_2017_214_MOESM2_ESM.pdf]

2015 - 2016

Certificate of Proficiency

This is to certify that

Room R4013
Childrens Hospital of Eastern Ontario
401 Smyth Road
Ottawa ON K1H 8L1

has participated in the international
25 Hydroxyvitamin D EQAS
and has met the performance target*
set by the DEQAS Advisory Panel

* 75% or more results fell within ±25% of the Target Value

Advisory Panel

Jacqueline Berry · Ramon A. Durazo-Arvizu
Elaine Gunter · Glenville Jones · Hugh Makin
Karen Phinney · Christopher Sempos

Graham Carter
DEQAS Organiser

VITAMIN D EXTERNAL QUALITY ASSESSMENT SCHEME

DEQAS
