# Supplementary material for: Study protocol for a phase II dose evaluation randomized controlled trial of cholecalciferol in critically ill children with vitamin D deficiency (VITdAL-PICU study)
Source: Pilot Feasibility Stud. 2017 Dec 8;3:70. doi: 10.1186/s40814-017-0214-z (PMC5721544; doi:10.1186/s40814-017-0214-z)
Supplement: Supplementary file 3 — Table. Description of minor protocol changes made during the conduct of the study (protocol versions 1 through 9). (PDF 82 kb) [file 40814_2017_214_MOESM3_ESM.pdf]

Additional File 3 Summary of modifications made to the VITdAL-PICU Study protocol since the initial REB approval

Protocol version number and date	Date of REB approval	Summary of modifications
Amendment #1, Protocol Version 2 (Version Date: 09-Dec-2015)	29-Feb-2016	1. Modification made to the DSMB Terms of Reference and protocol, as requested by the DSMB: (i) additional review added after the first 15 patients; (ii) time of review modified to when 15, 30 and 45 participants reach Day 7 of the study as opposed to following the initial study drug load; (iii) condition added to report all deaths and all requirements for renal replacement therapy to the DSMB; and (iv) option added for DSMB to review unblinded data at the DSMB meetings<br>2. A questionnaire to gather information on the type of research outcomes that are important for critically ill children and their caregivers created, and protocol modified to state it would be administered at enrolment.
Amendment #2, Protocol Version 3 (Version Date: 08-Mar-2016)	09-Mar-2016	1. The protocol was updated to state that the screening sample will either be measured using the Qualigen® FastPak or sent to a certified laboratory.<br>2. Additional recruitment procedures for cardiovascular surgery patients were added to protocol, since this patient group can be screened and approached for consent prior to PICU admission. Patients scheduled for cardiovascular surgery will be screened through the Cardiovascular Surgery clinic. If eligible, the study team will approach families for informed consent either at the pre-operative appointment, or on the day of surgery (prior to PICU admission).<br>3. In order to overcome challenges obtaining consent for eligible patients whose legal guardians do not come to the hospital during the day, and only visit in the evenings, as option was added to protocol to allow the study team to contact legal guardians of potential participants by telephone to introduce the study and to arrange a time to discuss the protocol and participation.<br>4. A clarification was made to safety protocol for patients whose last vitamin D level prior to discharge is >150 nmol/L. The protocol previously stated that Nephrology would follow all of these patients as outpatients, even if Nephrology did not have any safety concerns. We have clarified this process to state that all patients whose last vitamin D level prior to discharge is >150 nmol/L will be referred to Nephrology. Nephrology will then review their case and then decide if they should be followed as an outpatient to rule out any delayed symptoms of toxicity.
Amendment #3, Protocol Version 4 (Version Date: 27-May-2016)	06-Jul-2016	1. The study drug protocol was modified to eliminate administration of a second dose on Day 3, based on based on several factors, including: (i) a literature update suggesting that the second load may not be necessary, and evidence suggesting clinical benefit with a single enteral load [1, 2]; (ii) concern from other sites suggesting it may prevent their participation; (iii) cost of additional drug and measuring 25(OH)D status on Day 3 to determine dosing level; (iv) feedback from participants and potential participants that the study protocol, specifically the Day 3 dose, was complicated and difficult to understand which the authors felt may have a negative impact on recruitment rate; and (v) although procedures were put in place to maintain blinding during measurement of 25(OH)D at Day 3, the second dose increased the chances of accidental unblinding.<br>2. The length of PICU stay inclusion criteria was modified from an expected ICU length of stay of $\geq$3 days to an expected ICU length of stay of $\geq$2 days. Initially, this inclusion criterion was set at 3 days to ensure that study patients would still be in ICU at the time of the Day 3 dose, and to include patients who were seriously ill and likely to still be in hospital at Day 7 (time point for primary outcome). With the above-mentioned modification to the study drug protocol, this criterion was modified into 2 separate criteria: (i) expected PICU length of stay of $\geq$48 hours; and (ii) the treating physician expects that the patient will have access for bloodwork on Day 7 (i.e. patient will still be in hospital on Day 7 and likely to have clinical bloodwork).<br>3. The protocol was modified from a single-centre study to a multi-centre study with the addition of two international sites in Austria and Chile.<br>4. A modification was made to the safety process for patients whose last vitamin D level prior to discharge is elevated. Previously, the threshold to activate our safety protocol was >150 nmol/L. However, based on the 2015 Canadian Paediatric Society Position Statement on vitamin D supplementation, this threshold was increased to 200 nmol/L. Patients whose last 25(OH)D level prior to discharge is >200 nmol/L will now be referred to Endocrinology (as opposed to Nephrology).

		Endocrinology will review the patient's clinical course and blood ionized calcium levels and decide on the appropriate follow up actions.<br>5. A decision aid tool was created to assist families in making a decision about participating in the study.
Amendment #4, Protocol Version 5 (Version Date: 11-Oct-2016)	18-Oct-2016	1. A clarification was made to protocol to state that for randomized patients who are screened using discard blood, we will collect an enrolment sample prior to administration of study drug (discard blood has not been processed and stored properly and does not allow enough quantity for all of the proposed analyses). If a fresh sample is collected at the time of screening, the enrolment sample will not be collected. The amount of blood that will be collected from each patient still remains within the weight-based volume limits for blood draws imposed by REB.<br>2. Based on a preliminary analysis of responses to the research outcomes questionnaire (~80% of VITdAL-PICU patients and their families rated "quality of life following hospital discharge" as one of their top three most important research outcomes), the protocol was modified to capture pilot quality of life data using the PedsQL™. The questionnaire will be administered to patients (when appropriate) and their caregivers up to five times at the following time points: i) baseline, ii) PICU discharge, iii) hospital discharge, iv) Day 30, and Day 90. The information from this pilot quality of life data will be used during the design of grant applications and study protocol for the subsequent phases of this research program.<br>3. We have clarified the wording used when the Principal Investigator (PI) signs off on research results in order to more accurately capture the significance of research lab results. Results will be signed by the PI, dated, and indicated as being: (i) "below the study threshold"; (ii) "above the study threshold" if elevated but the patient has no clinical symptoms or concerns; or (iii) "clinically significant" if the result is elevated and the patient is experiencing symptoms potentially related to vitamin D.<br>4. An option was added to the protocol to re-screen patients in certain situations. If a patient is consented and the screening 25(OH)D level is >50 nmol/L (patient not eligible), but the patient subsequently undergoes an intervention known to reduce vitamin D levels (e.g. ECMO, bypass), a new blood sample (discard whenever possible) will be collected to re-screen the patient. If the 25(OH)D level in the post-intervention screening sample has fallen to <50 nmol/L, the patient will be randomized into the study. Additionally, if a screening 25(OH)D result is unexpected based on our experience with hundreds of ICU patients (either low or how), an additional screening sample may be collected and re-analyzed. This is the same process that is followed in clinical care for an abnormal laboratory result (new blood sample collected and re-analyzed). An abnormal sample result could suggest a problem with sample collection, processing, or performance of the Qualigen® assay. An abnormal 25(OH)D result was defined in the protocol as a result that is inconsistent with what is known about vitamin D levels in critically ill children.<br>5. Acknowledgement of financial support from CIHR has been added to the protocol.<br>6. Wording in the protocol regarding monitoring was clarified to state that a separate monitoring plan will be developed for international sites.
Amendment #5, Protocol Version 6 (Version Date: 31-Mar-2017)	7-Apr-2017	1. Procedures for telephone consent added to study protocol.
Amendment #6 Protocol Version 7 (Version Date: 10-Apr-2017)	24-Apr-2017	1. The sample size and DSMB review procedures were modified. Initially, the study drug protocol for VITdAL-PICU involved two doses: at enrolment and on Day 3. The study drug protocol was modified to a single dose part-way through recruitment. Seven patients received two doses of study drug before this modification was made. Our original sample size was 60 patients; however, following our recent Steering Committee meeting, the sample size was increased to 67 in order to account for the 7 patients who received two doses. The procedure for DSMB review was also modified. Instead of conducting the second DSMB review at 45 patients, the the review will now be conducted at 52 patients (45 + 7 patients) so that they can review safety data for 45 patients who enrolling using the single dose protocol. Note that the 7 patients who received two doses will still be included in the final intention to treat analysis but will not be included in the secondary per protocol analysis.

Amendment #7, Protocol Version 8 (Version Date: 04-May-2017)	25-May-2017	1.<br>2.	A simple permission form was developed and approved that can be given to legal guardians by the PICU care team, The form asks the legal guardian for permission to measure their child's vitamin D level in order to determine if their child is eligible for the study.<br>At the request of the REB at the Children's Hospital of Eastern Ontario, the telephone consent procedures were modified to state that telephone consent could only be obtained for determining eligibility (measurement of vitamin D status) but not trial participation.
Amendment #8, Protocol Version 9 (Version Date: 23-Aug-2017)	Pending	1.	Based on (i) the recent CPS statement regarding vitamin D levels[3], (ii) the Pediatric Endocrine Society recommendations[4], (iii) feedback from CHEO Endocrinology, and (iv) the recent change to the reference values for vitamin D at the CHEO Laboratory (sufficiency now defined as a 25(OH)D level of 75-249 nmol/L), the Stopping Rules in the study protocol and DSMB Terms of Reference were modified. Previously, the protocol and TOF stated that we would be concerned about the drug protocol and consider adjusting the dose if any study participant achieved 25(OH)D levels above 250 nmol/L or if more than 10% of study participants achieved 25(OH)D levels above 200, however, this was changed to state that we will consider modifying the dosing protocol if more than 10% of study participants achieved 25(OH)D levels above 250 nmol/L.

Abbreviations: DSMB – Data Safety Monitoring Board; PICU – Pediatric Intensive Care Unit; 25(OH)D – 25-hydroxyvitamin D; REB – Research Ethics Board; PedsQL™ - Pedatric Quality of Life TM; ECMO - Extracorporeal membrane oxygenation; CIHR – Canadian Institutes of Health Research; DSMB – Data Safety and Monitoring Board

1. Amrein K, Schnedl C, Holl A, Riedl R, Christopher KB, Pachler C, Urbanic Purkart T, Waltensdorfer A, Munch A, Warnkross H, et al: Effect of high-dose vitamin D3 on hospital length of stay in critically ill patients with vitamin D deficiency: the VITdAL-ICU randomized clinical trial. *JAMA.* 2014, 312:1520-30.
2. Han JE, Jones JL, Tangpricha V, Brown MA, Brown LA, Hao L, Hebbar G, Lee MJ, Liu S, Ziegler TR, Martin GS: High Dose Vitamin D Administration in Ventilated Intensive Care Unit Patients: A Pilot Double Blind Randomized Controlled Trial. *J Clin Trans Endo.* 2016, 4:59-65.
3. Godel JC: Vitamin D supplementation: recommendations for Canadian mothers and infants. *Paediatr Child Health.* 2007, 12:583-9.
4. Misra M, Pacaud D, Petryk A, Collett-Solberg PF, Kappy M, Drug, Therapeutics Committee of the Lawson Wilkins Pediatric Endocrine S: Vitamin D deficiency in children and its management: review of current knowledge and recommendations. *Pediatrics.* 2008, 122:398-417.
